# Selenite activates the alternative oxidase pathway and alters primary metabolism in *Brassica napus* roots: evidence of a mitochondrial stress response

**DOI:** 10.1186/s12870-014-0259-6

**Published:** 2014-09-30

**Authors:** Aleksandar Dimkovikj, Doug Van Hoewyk

**Affiliations:** Coastal Carolina University, Biology Department, Conway, SC 29526 USA

**Keywords:** Selenium, TCA cycle, Glutathione, Mitochondrial superoxide, γ-glutamyl cyclotransferase

## Abstract

**Background:**

Human requirements for dietary selenium are met mainly by crops. However, excessive uptake of selenium in plants can restrict growth, and its toxicity has been postulated to target roots. Selenite toxicity can be attributed to its assimilation into selenocysteine, which can replace cysteine to yield malformed selenoproteins. Additionally, selenite has pro-oxidant properties. In this study, the effects of selenite on root tissue in *Brassica napus* (canola) were investigated to better understand its mode of toxicity and the metabolic adjustments needed to mediate a selenite-response.

**Results:**

Selenite induced the rapid formation of mitochondrial superoxide, which led to decreased aconitase activity and involvement of the alternative oxidase pathway. Although selenite altered primary metabolism, as observed by the increased amino acids and decreased TCA cycle metabolites, increased glucose presumably supported higher respiratory rates and ATP levels reported in this study. Additionally, evidence is presented indicating that selenite suppressed the ubiquitin-proteasome pathway, and induced the pentose phosphate pathway needed to maintain antioxidant metabolism. Selenite treatment also elevated glutathione concentration and coincided with increased levels of γ-glutamyl cyclotransferase, which may possibly degrade selenium metabolites conjugated to glutathione.

**Conclusion:**

Collectively, the data indicate that selenite necessitates the reconfiguration of metabolic pathways to overcome the consequences of mitochondrial oxidative stress in root tissue. Efforts to mitigate the detrimental effects of selenite-induced oxidative stress may ultimately improve selenium tolerance and accumulation in crops.

**Electronic supplementary material:**

The online version of this article (doi:10.1186/s12870-014-0259-6) contains supplementary material, which is available to authorized users.

## Background

Higher plants are non-motile and must confront a variety of abiotic stressors in their environment that potentially restrict net primary production and development. A defining feature of abiotic stress in plants is the accumulation of reactive oxygen species (ROS). If a plant’s capacity to suppress ROS accumulation is overwhelmed or impaired, oxidative stress can ensue which can result in the oxidation of cellular macromolecules such as lipids, nucleic acids, and proteins [1]. In particular, mitochondrial proteins are prone to oxidation, as observed by the oxidative damage to subunits making up the pyruvate decarboxylase complex, NADH dehydrogenase complex, and ATP synthase in Arabidopsis cells [2]. Additionally, it is also well known that ROS can directly inhibit the iron-sulfur enzyme aconitase that participates in the tricarboxylic acid (TCA) cycle, which can ultimately lead to mitochondrial impairment [3]. The ability of plants to tolerate mitochondrial oxidative stress is governed by an effective response, including antioxidant machinery and the repair of damaged cellular components [4].

However, an oxidative stress response is energetically costly, and therefore requires metabolic fine-tuning. For example, maintenance of the glutathione-ascorbate cycle during oxidative stress is dependent upon NADPH production, which requires the redirection of sugars from glycolysis and into the oxidative pentose-phosphate pathway (OPPP), as observed in root tissue of Arabidopsis treated with menadione [5]; this study also noted that oxidative stress decreased levels of most TCA cycle metabolites, but resulted in amino acid accumulation, further suggesting that oxidative stress alters primary metabolism. Oxidative stress can also impose high-turnover costs to repair damaged molecules and organelles [6], and therefore force plants to allocate more sugars into respiration to maintain cellular homeostasis rather than direct the fixed carbon into growth. The metabolic costs associated with initiating a successful stress response are met by expenditure of ATP, which is made primarily by the mitochondrial electron transport chain. However, the effects of oxidative stress on plant respiration are conflicting, which likely reflects the severity of the stress (*i.e.* the respiratory response is time and dose-dependent). Heavy metals, for example, can both increase or decrease respiration in plants, as recently reviewed [7]. Whether or not a plant can meet the higher energetic costs required to combat oxidative stress is determined by metabolic adjustments that regulate respiratory potential. Therefore, respiration can fulfill a protective role during an oxidative stress response, and can dictate how well a plant can tolerate stress, such as salinity [8].

Recently it was reported that cadmium-treated Arabidopsis induced ROS accumulation in mitochondria prior to plastids [9]. This implies that mitochondria are not only targets of oxidative stress, but that they must act as sentinels and mediate the necessary signaling to initiate a response to ROS, as previously proposed [4,10]. During mitochondrial oxidative stress, the signaling molecule nitric oxide can help mediate cross-talk between mitochondrial aconitase and the alternative oxidase (AOX) pathway [11]. AOX has been implicated in a variety of stress responses, and although it does not contribute to the ATP pool, it alleviates over-reduction of the electron transport chain by redirecting electrons from Complex IV, thereby preventing the reduction of oxygen to superoxide [12]. Aconitase inhibition can activate the AOX pathway [11], and provides a mechanism explaining how mitochondrial superoxide can uncouple the TCA cycle and the downstream electron transport chain. This supports the increasing evidence that the AOX pathway maintains homeostasis of primary metabolism during stress [13,14].

This study examined the effects of selenite toxicity on primary metabolism in the roots of *Brassica napus*. Although higher plants do not have a requirement for selenium (Se), crops supply most of the dietary consumption of essential Se to humans and livestock. Additionally, *in vitro* studies have established the protective benefits of some Se metabolites against cancer [15]. Interest in plant Se metabolism stems from these studies, and Se-rich crops may be envisioned to help prevent disease or improve nutrition. *Brassica* crops, including *B. napus*, have demonstrated potential for their ability to accumulate Se [16].

However, efforts to create Se-fortified crops are be restricted to plants’ ability to respond to and tolerate Se toxicity, which has been shown to target Arabidopsis roots [17]. Selenite stress is known to cause two distinct types of stress. Thus, elucidating of the effects of selenite toxicity in roots might better serve efforts to augment Se tolerance or accumulation in crops. One mode of selenite toxicity occurs when it is assimilated into selenocysteine, which can then randomly replace cysteine in protein [18]; the resultant selenoprotein is likely malformed, and can be targeted for removal by the ubiquitin-proteasome pathway in the leaves of *Stanleya pinnata* [19]. Additionally, selenite is a pro-oxidant that can induce oxidative stress and the accumulation of ROS in a wide-range of plants, as recently reviewed [20]. In plants, selenate can be reduced to selenite enzymatically; however, the subsequent non-enzymatic reduction of selenite is likely mediated by glutathione [21], which is known to generate superoxide [22]. Recently, human cells treated with selenite induced the accumulation of mitochondrial superoxide and rapidly changed mitochondrial morphology [23]. However, it is not known if selenite similarly results in mitochondrial superoxide accumulation in plants. Given that the concentration of GSH in plants is highest in mitochondria [24], we reasoned that selenite would generate mitochondrial superoxide in *B. napus* roots and likely impact respiration and primary metabolism. Thus, the objective of this study was to better understand the metabolic adjustments that occur in roots in response to selenite stress. The data strongly indicate that selenite induced mitochondrial stress, as observed by the accumulation of mitochondrial superoxide and activation of the AOX pathway. Selenite had antagonistic effects of TCA cycle metabolites and amino acids, yet ATP levels increased. The importance of metabolic adjustments in response to Se stress is discussed in view of the evidence that selenite alters the energetic demands in *B. napus*.

## Results

Preliminary work examined the effects of 0, 20, 50, 100, and 250 μM selenite after three days of treatment. A concentration of 50 μM was selected to further study the short term effects of selenite toxicity, because it reduced root growth without severely affecting the fresh weight to dry weight ratio of the roots and root cell viability (Additional file 1: Figure S1). In contrast, 250 μM selenite greatly reduced cell viability and altered the fresh weight to dry weight ratio.

Prior to focusing on the short-term effects of selenite in root tissue, initial experiments were centered on establishing the physiological effects of 50 μM selenite before the visible onset of necrosis or chlorosis in *B. napus* plants. To meet this challenge, *B. napus* were treated with selenite for 7 days. After a week of treatment, net primary productivity and root growth decreased nearly two- and three-fold, respectively, in selenite-treated plants compared to untreated plants (Additional file 2: Figure S2a,b). Despite the effects of selenite on growth, there was no difference in photosynthetic parameters (Additional file 2: Figure S2c,d), including chlorophyll content and Fv/Fm values in dark-adapted plants, which reflects the optimal efficiency of photosystem II. Although selenite did not affect chlorophyll content, it increased the concentration of the pigment anthocyanin, which can prevent photoinhibition during stress (Additional file 2: Figure S2e). Lastly, fluorescent microscopy revealed that selenite resulted in the production of ROS in root tissue (Additional file 2: Figure S2f), as determined by using the fluorescent probe 2’ ,7’-dichlorodihydrofluorescein diacetate (H2DCFDA).

To confirm that selenite stress was caused by its accumulation in plant tissue, the elemental content of root and leaf tissue was determined after one week of selenite treatment (Additional file 3: Figure S3). As expected, total Se accumulated in the roots of plants treated with selenite but was not easily translocated to leaf tissue. Selenite-treatment increased the amount of total sulfur in root tissue, but intriguingly decreased the concentration of total sulfur in leaves.

### Se induced mitochondrial superoxide accumulation and decreased aconitase activity

**Figure 1:**
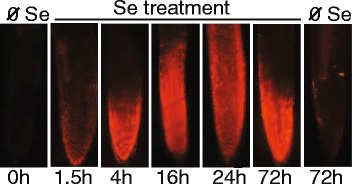
**Selenite induces the accumulation of mitochondrial superoxide.** Three-week old *B. napus* plants were treated with 50 μM selenite for the time indicated, at which point root tips were excised and incubated with the fluorescent probe MitoSox. Epifluorescence of Mitosox was representative of 8–10 root tips from 5 different plants.

As noted above, selenite treatment for 7 d induced the accumulation of ROS in root tissue. Reactive oxygen species are produced primarily in plastids and mitochondria, the latter containing a higher concentration of GSH. Given that the GSH mediated-reduction of selenite to selenide generates superoxide, it was hypothesized that selenite-treatment might stimulate the accumulation of mitochondrial superoxide. The fluorescent probe MitoSox Red (Invitrogen), which specifically fluoresces in the presence of mitochondrial superoxide [25] and is restricted from plant plastids [12,26], was used to determine if selenite induces the accumulation of mitochondrial superoxide. Mitosox fluorescence is observed after 1.5 h of selenite-treatment; by 16 hours the fluorescence extends throughout the root tip, and is still evident on day 3 (Figure 1).

**Figure 2:**
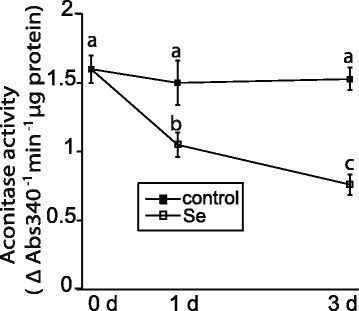
**Selenite decreases aconitase activity.** Aconitase acitivity in root tissue was measured from untreated plants and plants treated with 50 μM selenite for 1 and 3 days. Shown are the mean and SE from 5 different plants. Lowercase letters represent a significant difference between treatments (p < 0.05).

The effects of selenite on primary metabolism in root tissue were further investigated 1 and 3 d after treatment. The accumulation of mitochondrial superoxide can impair protein function, including aconitase, and potentially alter respiration. Selenite-treatment on d 1 and 3 decreased aconitase activity nearly 30% and 50%, respectively, compared to untreated plants (Figure 2). Thus, the accumulation of mitochondrial superoxide on d 1 and 3 coincides with a decrease in aconitase activity in Se treated plants.

**Figure 3:**
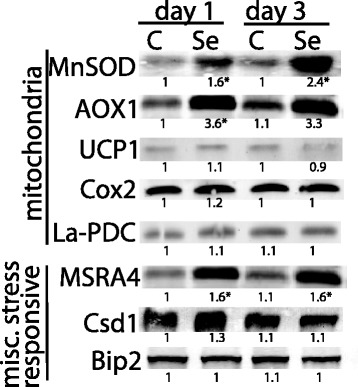
**The effect of selenite treatment on the accumulation of polypeptides in** ***B. napus*** **.** Immunoblot of mitochondrial and stress response proteins in root tissue from untreated plants and plants treated with 50 μM selenite for 1 and 3 days. 20 ug of denatured protein was loaded per lane and analyzed on SDS-PAGE. The immunoblot is representative of at least three biological experiments, and numbers below each blot represent the mean pixel intensity of each immunoreactive band relative to day 0. Asterisks indicate a significant difference in band intensity in selenite-treated plants compared to untreated plants (p < 0.05).

The levels of mitochondrial proteins and stress-responsive proteins were analyzed to further determine the impact of selenite on root tissue after d 1 and 3 (Figure 3). Se increased levels of manganese superoxide dismutase (MnSOD), a mitochondrial protein that quenches superoxide. Mitochondrial superoxide accumulation can also be alleviated by the AOX pathway, which diverts electrons away from the cytochrome c oxidase (COX) pathway. Protein levels of AOX1 increased dramatically (>3-fold) after selenite treatment for 1 and 3 d. In contrast, an uncoupling protein (UCP1) that can help dissipate the mitochondrial proton gradient was not affected by selenite. Protein levels of COX2, a subunit of the COX complex (complex IV), also remained relatively unchanged. Another marker for mitochondrial oxidative stress is a decrease in the lipoic acid bound to the E2 subunit of the pyruvate dehydrogenase complex [2], which was intriguingly unaffected by Se. In addition to mitochondrial proteins, selenite induced the accumulation of a plastid-localized methionine sulfoxide reductase (MSRA4); this protein repairs oxidized methionine residues that result from oxidative stress [27]. Selenite did not significantly increase the level of CSD1, the cytosolic copper-zinc SOD. Similarly, Se did not impact levels of the ER protein Bip2, which participates in protein folding and accumulates during the unfolded protein response during ER stress.

### Selenite alters primary metabolism and respiration

The effects of selenite on primary metabolism were investigated by measuring TCA cycle intermediates and amino acids on day 3. A targeted metabolic analysis indicated that selenite treatment decreased many of the metabolites produced during the TCA cycle d 3 compared control (Table 1). The effect of selenite was most pronounced on levels of metabolites produced during later steps of the TCA cycle, including succinate and fumarate, which decreased 4-fold compared to untreated roots. Intriguingly, levels of oxoglutarate, which links the TCA cycle to glutamate synthesis, increased on d 3. Additionally, levels of pyruvate- which connects glycolysis to the TCA cycle- doubled on d 3. Although selenite decreased most TCA cycle metabolites on day 3, it increased the levels of all amino acids analyzed (Table 2). Most notably, alanine accumulated 6-fold and levels of cysteine, which is used to make glutathione, increased 4-fold during selenite treatment.

**Table 1:** **The effect of selenite on TCA cycle metabolites and ascorbate**

	**Control**	**Selenite**
pyruvate	1 (0.08)	2.22 (0.25)*
citrate	1 (0.06)	0.55 (0.03)*
isocitrate	1 (0.05)	0.59 (0.06)*
aconitate	1 (0.07)	0.86 (0.08)
oxoglutarate	1 (0.09)	1.63 (0.08)*
succinate	1 (0.02)	0.20 (0.04)*
fumarate	1 (0.08)	0.22 (0.04)*
malate	1 (0.08)	0.45 (0.03)*
ascorbate	1 (0.12)	0.88 (0.14)

The relative levels of metabolites were measured in untreated plants (day 0) and plants treated with selenite for 3 days. Values represent levels relative to control, and are the mean and SE of 4 replicates from individual plants. Asterisks denote a significant difference between treatments (p < 0.05).

**Table 2:** **The effect of selenite on amino acid and ammonia concentration in root tissue**

	**Control**	**Selenite**
asp	1.0 (0.02)	2.4 (0.14)^*^
thr	1.0 (0.05)	1.6 (0.09)^*^
ser	1.0 (0.05)	2.1 (0.06)^*^
glu	1.0 (0.03)	2.7 (0.16)^*^
gln	1.0 (0.04)	3.1 (0.14)^*^
gly	1.0 (0.07)	1.6 (0.05)^*^
ala	1.0 (0.04)	6.4 (0.33)^*^
val	1.0 (0.06)	2.5 (0.11)^*^
cys	1.0 (0.04)	4.5 (0.09)^*^
met	1.0 (0.05)	1.5 (0.04)^*^
lys	1.0 (0.04)	3.1 (0.05)^*^
his	1.0 (0.04)	2.4 (0.04)^*^
arg	1.0 (0.02)	2.0 (0.10)^*^
GABA	1.0 (0.06)	6.1 (0.22)^*^
NH3	1.0 (0.06)	1.7 (0.12)^*^

The relative levels of free amino acids (nmol −1 100 mg FW) were measured in root tissue from untreated plants and plants treated with selenite for 3 days. Values represent levels relative to control, and are the mean and SE of 4 replicates from individual plants. Asterisks denote a significant difference between treatments (p < 0.05).

**Figure 4:**
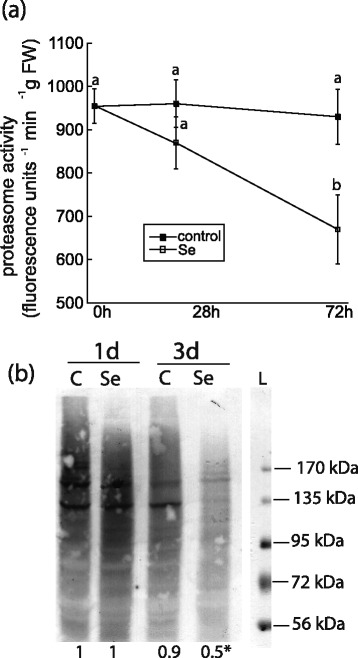
**The effects of selenite on the ubiquitin-proteasome pathway in root tissue. (a)** Proteasome activity in roots of untreated plants and plants treated with 50 μM selenite for 1 and 3 d. Shown are the mean and SE in 5 different plants. Values represent fluorescence of proteasomally-released AMC at the each time interval. Data are the mean of three biological replicates and standard deviation. Lowercase letters represent a significant difference in activity at each time point (*p* < 0.05). **(b)** The accumulation of high-molecular weight ubiquitinated proteins in the roots of *B. napus* from untreated plants and plants treated with 50 μM selenite for 1 and 3 days, and then supplemented in 0.1% DMSO with or without 100 μM MG132 in for 8 hours. 50 μg of protein were separated on an 8% SDS gel, and ubiquitinated proteins were detected using anti-ubiquitin antiserum. The immunoblot is representative of at least three biological experiments, and numbers below each blot represent the mean pixel intensity of all the immunoreactive bands relative to control on day 1. Asterisks indicate a significant difference in band intensity in selenite-treated plants compared to untreated plants (p < 0.05). L = ladder.

The ubiquitin-proteasome pathway, which can selectively degrade proteins to fuel respiration during nutrient deprivation [28], was examined to determine the possibility that it contributed to the increased amino acid pool during Se treatment. However, proteasome activity was unaffected by Se on d 1 and decreased nearly 30% by d 3 (Figure 4a). The accumulation of high-molecular weight poly-ubiquitinated proteins was also measured in proteasome-inhibited MG132-treated plants. On d 3, selenite reduced the abundance of ubiquitinated proteins by 40% (Figure 4b), and coincides with reduced proteasome activity.

**Figure 5:**
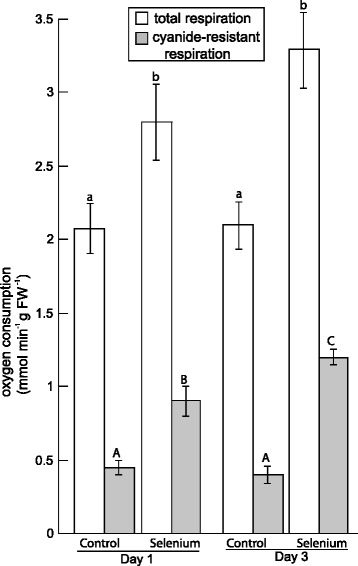
**Selenite stimulates the alternative respiratory pathway.** Total respiration and cyanide-resistant respiration, which is indicative of the AOX pathway, were estimated by measuring the rate of oxygen consumption in root tissue from untreated plants and plants treated with 50 μM selenite for 1 and 3 d. Shown are the mean and SE from 8 different plants per treatment. Lowercase and uppercase letters represent significant differences in total respiration and cyanide-resistant respiration, respectively (p < 0.05).

As mentioned above, selenite induced the abundance of the AOX1 protein. AOX is known to be post-translationally regulated, and thus the protein levels and activity of AOX can be uncoupled [29]. Therefore, it was desirable to determine if selenite altered respiration by increasing the flux of electrons donated to the cyanide-resistant AOX pathway. Total respiration and cyanide-resistant respiration, which is indicative of AOX activity, were measured by determining the oxygen-consumption in root tissue of plants with or without Se. Selenite increased cyanide-resistant respiration 2- and 3-fold by d 1 and d 3, respectively (Figure 5). Although oxygen consumption of the cytochrome c oxidase pathway was not directly measured, total respiration also increased on d 1 and 3 of selenite treatment compared to untreated samples on d 0.

**Figure 6:**
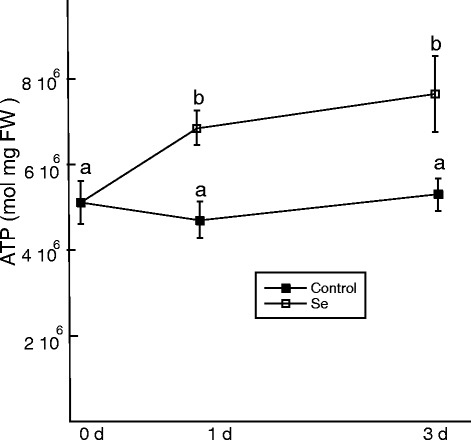
**The effect of selenite on ATP levels.** ATP was measured in root tissue from untreated plants and plants treated with 50 μM selenite on d 1 and 3. Shown are the mean (n = 8 individual plants) and SE of. Lowercase letters represent significant differences between treatments (p < 0.05).

The antagonistic effect of selenite on TCA cycle metabolites and respiration warranted analysis of ATP and upstream sugars to determine the effect of Se on the energy budget and carbohydrate status of the roots. After d 1 and 3, levels of ATP in root tissue increased roughly 1.5-fold when challenged with selenite (Figure 6).

**Figure 7:**
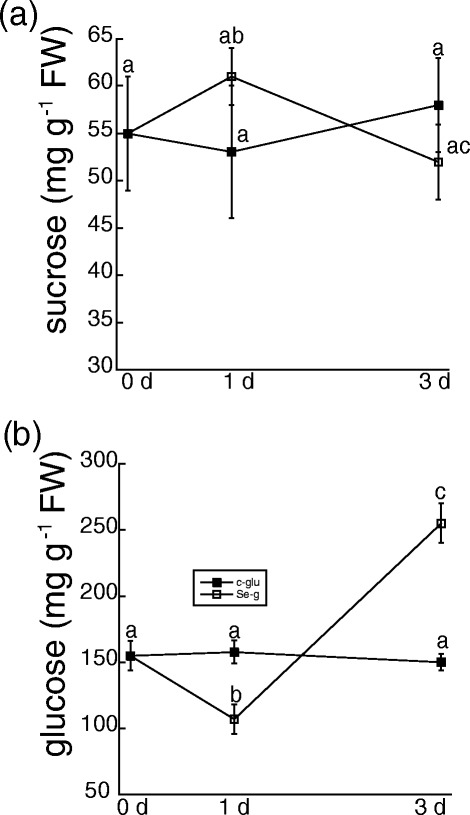
**The effect of selenite on soluble sugars.** Sucrose **(a) **and glucose **(b)** were estimated enzymatically from the roots of untreated plants and plants treated with 50 μM selenite for 1 and 3 d. Shown are the mean and SE from 6 individual plants. Lowercase letters represent significant differences between treatments (p < 0.05).

Selenite did not affect sucrose concentration (Figure 7a). In contrast, selenite decreased glucose by 30% on d 1, but then almost doubled in concentration on d 3 compared to d 0 (Figure 7b).

### Se increases GSH and γ-glutamyl cyclotransferase

**Figure 8:**
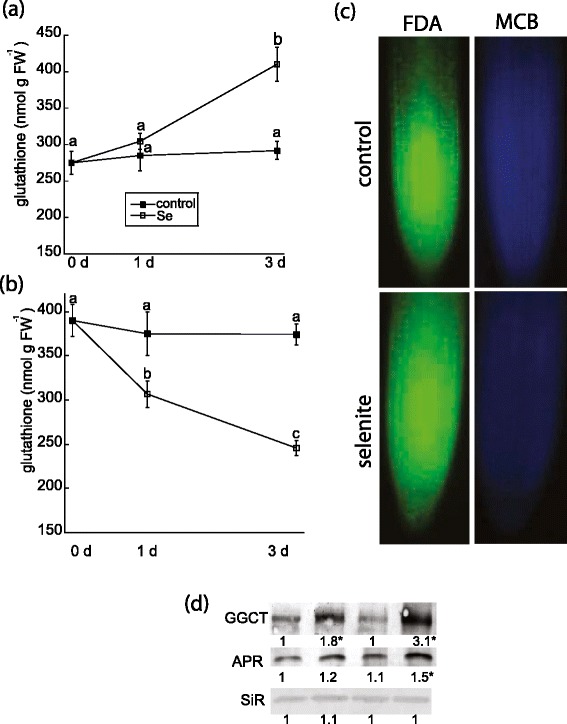
**The effect of selenite on glutathione metabolism.** The levels of glutathione were measured enzymatically in total root tissue **(a)** and root tips **(b)** 8–12 mm in length from untreated plants and plants treated with 50 μM selenite for 1 and 3 d. Shown are the mean and SE from 4 different plants, and represent 2 other biological experiments. Lowercase letters represent significant differences among treatments (p < 0.05). **(c)** Cell viability and glutathione content in root tips were estimated as the fluorescence of fluorescein diacetate (left) and monochlorobimane (right), respectively from 8–10 pooled root tips from 4 plants per treatment on day 3. **(d)**. Abundance of the putative GGCT, APR, and SiR polypeptides were estimated by loading 20 μg of denature protein from root tissue per lane on SDS-PAGE and analyzed by immunoblotting. The immunoblot is representative of at least three biological experiments, and numbers below each blot represent the mean pixel intensity of each immunoreactive band relative to control on day 0. Asterisks indicate a significant difference in band intensity in selenite-treated plants compared to untreated plants (p < 0.05).

As previously mentioned, selenite induced the accumulation of ROS, which can be quenched by the NADPH-dependent glutathione-ascorbate cycle. Glutathione is known to be critical to oxidative stress tolerance in plants, and thus, the effect of selenite on the metabolites and pathways that maintain the glutathione-ascorbate cycle were investigated. Metabolite analysis indicated that selenite did not affect levels of ascorbate (Table 1). In contrast, glutathione in root tissue increased 1.5-fold by d 3, but there was no difference in glutathione content 1 d after selenite treatment (Figure 8a). Maintenance of a high concentration of GSH is critical for root growth in Arabidopsis [30]. Because Se restricted root length in our study, GSH content in root tips were analyzed. In contrast to total root tissue, Se decreased GSH concentration in root tips on d 1 and 3 by nearly 20 and 30%, respectively (Figure 8b). Microscopy confirmed that Se decreased GSH, as estimated by the fluorescence of monochlorobimine in root tips treated with Se for 3 d. However, cell viability estimated by fluorescence of fluoresceine diacetate was not affected by Se-induced GSH depletion in roots tips (Figure 8c).

Optimal glutathione metabolism in Arabidopsis plants subjected to heavy metal stress is maintained by the newly discovered γ-glutamyl cyclotransferase (GGCT2; 1); this enzyme participates in the γ-glutamyl cycle by recycling glutamate from GSH-conjugates, which can subsequently be used to make glutathione [31]. The predicted GGCT2;1 protein in *B. rapa,* a close relative to *B. napus,* shares 94% sequence similarity to the Arabidopsis homologue (Additional file 4: Figure S4). In *B. rapa*, the predicted GGTC2;1 protein contains 220 amino acids, and its paralogous protein GGCT2;2 contains 223 amino acids. Protein levels of GGCT in *B. napus* roots were examined to determine if its abundance is affected by selenite. The immunoblot reveals that selenite rapidly induced the accumulation of GGCT, as revealed by a single band that is approximately 25 kDa (Figure 8d), which is close to the nearly identical size of GGCT2;1 and GGCT2;2 in *B. rapa*. Therefore, selenite increases levels of GGCT, an enzyme that plays a role in glutathione biosynthesis during stress. Levels of proteins regulating the sulfur assimilatory pathway leading to cysteine and eventual glutathione biosynthesis were also analyzed to determine if they are affected by selenite. On d 3, selenite increased the abundance of adenosine 5-phosphosulfate reductase (APR), a rate-limiting enzyme in sulfur assimilation that reduces activated sulfate to sulfite. In contrast, selenite did not affect levels of sulfite reductase (SiR).

### Se stimulates the OPPP

**Figure 9:**
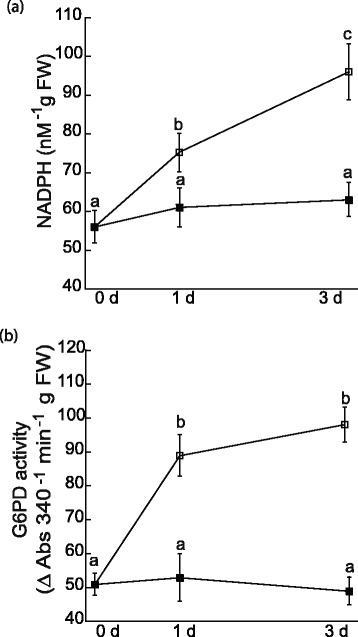
**Selenite increases levels of NADPH and activity of G6PD.** Levels of NADPH **(a)** and glucose-6-phosphate dehydrogenase (G6PD) activity **(b)** were measured in root tissue from untreated plants and plants treated with 50 μM selenite on day 0, 1, and 3. Shown are the mean and SE of 6–8 plants. Lowercase letters represent significant differences between treatments (p < 0.05).

The glutathione-ascorbate cycle is maintained by the reductant NADPH, which reduces oxidized glutathione. Levels of NADPH increased 1.3- and 1.6-fold in the root tissue of plants subjected to selenite stress after d 1 and 3, respectively (Figure 9a). In root tissue, NADPH is produced by glucose-6-phoshphate dehydrogenase, a cytosolic enzyme in the oxidative pentose-phosphate pathway (OPPP) that diverts glucose from glycolysis. The activity of glucose-6-phosphate dehydrogenase was measured to determine if the increased concentration of NADPH during selenite treatment was a consequence of increased partitioning of sugars into the OPPP or rather decreased consumption of NADPH. Activity of glucose-6-phoshphate dehydrogenase nearly doubled 1 and 3 d after selenite treatment compared to untreated samples (Figure 9b).

## Discussion

In this study, the short-term effect of selenite-induced oxidative stress is directly investigated in root tissue, which has previously been postulated to be the primary target of selenium toxicity [17]. Although we report the effects of selenite on metabolic processes in roots, the role of mitochondria in alleviating selenite toxicity was previously foreshadowed in Arabidopsis expressing a broccoli methyltransferase involved in ubiquinone biosynthesis; these transgenic plants had increased selenite tolerance, which was associated with decreased ROS [32]. Ubiquinone is involved in the mitochondrial electron transport chain, and also has antioxidant properties that can decrease mitochondrial superoxide and protect respiration in human cells during stress [33]. Thus, elevated levels of ubiquinone- a mitochondrial metabolite- mitigated the effects of selenite-induced oxidative stress [32], and supports our findings that selenite toxicity targets mitochondria.

### Selenite has antagonistic effects on TCA cycle metabolites and amino acids

Selenite-induced oxidative stress altered the TCA cycle after 3 days as judged by a decrease in aconitase activity and TCA cycle metabolites. With the exception of oxoglutarate, Se decreased all other TCA cycle metabolites, most notably succinate and fumarate. The accumulation of pyruvate, which connects glycolysis to the TCA cycle, further suggests that the TCA cycle was impaired.

Intriguingly, however, Se resulted in the accumulation of amino acids, an observation that closely mirrors previous studies in heavy metal treated plants [34] as well as selenium-treated Arabidopsis [35,36]. Sulfur (which increased during Se treatment) and nitrogen metabolism are co-regulated, and incorporating excessive nitrogen into amino acids likely prevents the toxic accumulation of ammonia [37] or potentially serves as a source of energy during Se treatment. The disparate effects of selenite on TCA metabolites and amino acids in *B. napus* roots is reminiscent of another study investigating the short-term effects of oxidative stress on Arabidopsis roots [5]; in that study, menadione also decreased many TCA cycle metabolites, but increased levels of oxoglutarate, many amino acids, and pyruvate. The accumulation of amino acids during Se treatment may be regulated by the AOX pathway. Pyruvate and NADPH can post-translationally regulate the AOX pathway [29], which can in turn increase levels of amino acids during oxidative stress [11]. Collectively, these previous results are in agreement with our study, *i.e.* increased alternative respiration and the concomitant increase in amino acids may be regulated by higher levels of pyruvate and NADPH in Se treated plants. However, it should be noted that regulation of AOX was not directly studied, and that increased cyanide-resistant respiration likely stems from higher levels of the AOX1 protein observed in our study.

Selenite induced signatures of a mitochondrial stress response, but this does not necessarily indicate respiratory impairment as much as it requires metabolic adjustments. Supporting this assumption is the observed increase in ATP and total respiration, despite evidence of TCA impairment. Uncoupling of TCA inhibition and respiration was also reported in transgenic Arabidopsis with decreased MnSOD levels [10]; this was explained by a cytosolic bypass of a damaged TCA cycle. In our study, the direct effects of selenite on the TCA cycle are difficult to gauge because we examined root tissue and not isolated mitochondria. However, if selenite indeed impaired the TCA cycle, increased respiration could still be achieved as a result of external NADH dehydrogenases, TCA flexibility through the GABA shunt or rerouting of sugars through fermentative pathways. Re-examination of a transcriptome study in root tissue of Arabidopsis treated with selenate supports these possibilities [36]. The microarray data demonstrate that Se increased the glutamate dehydrogenase transcript 11-fold in roots; this protein regulates carbon and nitrogen metabolism in roots and bypasses damaged aconitase to provide the TCA cycle with oxoglutarate [38], which was the only TCA cycle metabolite that increased in our study. Additionally, the microarray data indicated that Se up-regulated two dicarboxylic carriers in root tissue, a common signature of a mitochondrial stress response [39]; these carriers bring redox equivalents into the mitochondria for NADH production [40]. Lastly, upregulation of two pyruvate decarboxylases and alcohol dehydrogenase in Arabidopsis root tissue possibly suggests that Se stimulates the fermentative pathway to generate optimal ATP levels [36]. In conclusion, the transcriptome study in Arabidopsis reaffirms the necessity of metabolic plasticity that is a consequence of Se stress.

### Selenite reconfigures primary metabolism to meet the energetic demands associated with selenite-induced oxidative stress

Although Se is not essential to higher plants, in recent years the growth-stimulatory effects of selenium at low concentrations have been well-documented and reviewed [41]. In our study, selenite-treatment increased respiration and glucose levels after 3 days, but clearly it did not improve growth. Abiotic stress can cause the accumulation of glucose in plants [42], which can either fuel respiration or signal an ROS response, including activation of cytosolic glucose-6-phosphate dehydrogenase [43]. Selenite increased NADPH levels and activity of glucose-6-phosphate dehydrogenase, suggesting an increased flux of glucose through the OPPP to maintain the glutathione-ascorbate cycle [5]. Additionally, the turnover of oxidized proteins caused by Se [19] and antioxidant metabolism is energetically expensive [6], which is likely why ATP levels were elevated. Taken together, our data suggest that sugar was redirected from growth to fuel respiration and the OPPP in order to maintain cellular homeostasis during Se stress.

### Mitochondrial superoxide may impair the ubiquitin-proteasome pathway in plants

Although the 26S ubiquitin-proteasome pathway can remove misfolded proteins that tend to accumulate during stress, selenite decreased levels of ubiquitinated proteins and proteasome activity on day 3, and rules out the likelihood that the increased amino acids are a result of proteasomal degradation to fuel respiration [28]. Decreased proteasome activity has also been observed in sunflower plants subjected to a variety of different metals [44]. In human cells the accumulation of ubiquitinated proteins and proteasome activity is inhibited by mitochondrial superoxide, which directly impairs the activity of E1 ubiquitin activating and E2 ubiquitin conjugating enzymes [45]. Thus, it is feasible that the decreased proteasome activity and ubiquitinated proteins in Se-treated plants on d 3 could reflect an accumulation of mitochondrial superoxide, although this has yet to be experimentally determined.

### γ-glutamyl cyclotransferase is implicated in a selenite-response

Elevated GSH status is associated with improved oxidative stress tolerance in plants [46], and the increase in GSH in *B. napus* root tissue likely aids in the oxidative-stress response. Increased GSH concentration during Se treatment is concomitant with elevated protein levels of a putative γ-glutamyl cyclotransferase (GGCT) in *B. napus*. This cytosolic protein can maintain glutamate and GSH homeostasis during stress by mediating the breakdown of xenobiotics conjugated to GSH [31]. Overexpression of the GGCT2;1 protein in Arabidopsis improved arsenic tolerance, which was explained by the increased cytosolic breakdown of GSH conjugated to arsenic which lead to higher glutamate levels needed to maintain GSH in plastids and possibly mitochondria; it also decreased demand of *de novo* glutamate synthesis generated by the TCA cycle. Whether or not GGCT is involved in the breakdown of selenodiglutathione or Se metabolites conjugated to GSH is not known. Nonetheless, the accumulation of the GGCT protein points to its involvement in a Se-stress response. This conclusion is also supported by a 95-fold up-regulation of the transcript encoding GGCT2; 1 in root tissue of Arabidopsis plants treated with selenate [36].

### Metabolic alterations may underpin Se tolerance in plants

Although Se stress has been linked to decreased photosynthetic capacity in wheat [47], our study demonstrated that selenite altered primary metabolism in *B. napus* without affecting photosynthetic capacity after 7 days. Therefore, it is questionable if long-term selenium stress and growth impairment in crops is more attributable to photosynthetic damage or rather the increased flux of sugars into respiration and the OPPP to satisfy plants’ ATP and NADPH quota for maintenance costs associated with oxidative stress. Se-tolerance in cultivars of wheat [48] and ryegrass [49] are associated with its antioxidant capacity, which is regulated by mitochondrial processes [50]. In this context it is worth noting that *Brassica* species are generally considered as Se accumulators that are more Se-tolerant compared to Arabidopsis, wheat, and ryegrass [18,51]. Whether or not the range of selenium tolerance in plants is governed by differential adjustments to primary metabolism isn’t known. However, previous studies investigating plants’ comparative stress tolerance to copper [52] and salinity [53] have noted the importance of metabolic alterations, including respiration. Future studies aimed at discerning metabolic alterations between Se-sensitive and Se-tolerant plants may provide insight on how to improve Se tolerance in crops.

## Conclusions

**Figure 10:**
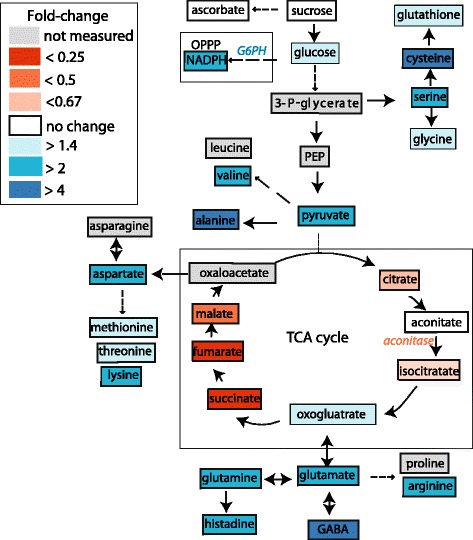
**Selenite reconfigures primary metabolism in root tissue of B. napus.** A schematic diagram illustrates the global effects of selenite treatment on metabolism after day 3. Each box represents the fold-change in metabolite abundance in selenite-treated roots relative to untreated roots. Dotted lines represent more than one enzymatic step. Relative differences in enzymatic activity from selenite-treated roots are italicized.

Several lines of evidence indicate that selenite caused mitochondria stress and metabolic adjustments in roots, as summarized in Figure 10. Selenite resulted in the rapid accumulation of mitochondrial superoxide and was accompanied by inhibition of aconitase activity, a known target of superoxide. Additionally, Se treatment increased levels of AOX1 and cyanide respiration, thus invoking the AOX pathway, which suppresses mitochondrial superoxide accumulation and provides metabolic poise during stress. Therefore, selenite can now be added to the growing list of abiotic stressors that involve the AOX pathway in a stress response.

A selenite response in *B. napus* necessitates the reconfiguration of primary metabolism, as determined by the increased levels of amino acids and the decrease in many TCA cycle metabolites. The increased respiratory rates and ATP levels highlight the importance of metabolic plasticity needed to support antioxidant metabolism during Se stress. This conclusion is supported by the observed increase in glutathione, as well as the increase in NADPH levels and activity of glucose-6-phosphate dehydrogenase, a key enzyme in the OPPP that maintains the glutathione-ascorbate cycle during oxidative stress. In summary, mounting a selenite-stress response in roots is energetically costly, and requires fine-tuning of primary metabolism. It is possible that differential metabolic adjustments may underpin Se tolerance in crops.

## Methods

### Growth condition and initial stress-induced measurements

*Brassica napus* plants were germinated in a growth chamber (250 μmol m^−2^ s^−1^ PAR, 14 h light/10 h dark cycle, 24°C) prior to being transferred after 7 d to Hoagland’s media consisting of 2 mM MgSO_4_. The roots of 16–20 plants were constantly aerated in 4 L of Hoagland’s media, which was changed every 5–7 days. After three weeks of growth in hydroponic media, the plants were treated with or without 50 μM sodium-selenite. Preliminary experiments focused on the effects of Se after 7 d to confirm that Se resulted in stress. The effects of Se on net primary productivity and root growth were determined as the difference of plant weight and root length, respectively, at day 0 and then either day 3 or day 7 after Se treatment in 15 different plants. Analysis of the pigments chlorophyll and anthocyanin, as well as the Fv/Fm ratio in dark adapted plants, were estimated in 10 plants on days 0 and 7 as previously described [35]. Elemental analysis of dried leaf and root tissue from 4 different plants treated without or with Se for 7 days were analyzed by ICP-MS (inductive coupled plasma-mass spectroscopy) at North Carolina State University, USA. For all remaining experiments, unless otherwise described, root tissue was harvested from individual plants with or without Se treatment on day 0, 1, and 3.

### Metabolite analysis

For the estimation of free amino acids and ammonia, 150 mg of root tissue from 4 separate plants harvested on day 3 were ground in liquid nitrogen, and extracted in 600 μL of water:chloroform:methanol mixture (3:5:12 v/v ratio). After centrifugation at 16,000 rpm, the upper water:methanol phase was lyophilized and analyzed on a Hitachi-L amino acid analyzer. For the targeted estimation of ascorbate, glutamate, pyruvate, and TCA cycle metabolites, 20 mg of root tissue were ground in liquid nitrogen and extracted in 1 mL of 70% methanol. Relative quantification of targeted metabolites was estimated using a gas-chromatography mass-spectroscopy at Colorado State University, USA.

Sucrose and glucose were determined using a sucrose assay kit and glucose assay kit, respectively (Sigma-Aldrich, USA), from the roots of 6 individual plants for each treatment. Glucose was estimated in a couple-enzyme assay containing hexokinase and glucose-6-phosphate dehydrogenase; during this reaction, NAD is reduced to NADH, which was measured spectrophotometrically (A_340nm_). Sucrose concentration was determined by first allowing the disaccharide to react with invertase, which yielded the products fructose and glucose. The glucose created from the invertase-mediated reaction was estimated as the reduction of NAD to NADH as described above. The sucrose concentration was measured as the difference in absorbance between the reactions with and without invertase.

ATP in root tissue was estimated using an ATP bioluminescence kit (Promega, USA). ATP was extracted in 1 mL of 3% trichloroacetic acid by grinding tissue (150 mg) in liquid nitrogen. After centrifuging (16,000 rpm), 300 μL of supernatant was neutralized in potassium-hydroxide (pH 7.8). ATP was estimated by adding 5 μL of extract into 95 μL of reaction buffer containing luciferase, and luminescence was immediately measured on a Promega Glo-Max Multi Jr luminometer.

Measurements of NADPH was performed as described [49], with some modifications. Briefly, 100 mg of root material from 8 different plants were extracted in 1 mL of 0.2 N NaOH by grinding in liquid nitrogen. Samples were centrifuged (16,000 rpm) for 10 m at 4 C, at which point 200 μL of supernatant was boiled for 1 m, rapidly cooled on ice, and then neutralized to pH 7.5 by adding 163 μL of 0.2 N HCL to 200 μL of the supernatant. NADPH was estimated as the NADP-dependent activity of glucose-6-phosphate dehydrogenase. For this reaction, 30 μL of neutralized extract was added to the reaction buffer containing 50 mM potassium-phosphate buffer, pH 7.5, 2 mM EDTA, 20 μL of 1.2 mM 2,6-dichlorophenolindophenol, 10 μL of 1 mM phenazine methosulfate, and 10 μL of 10 mM glucose-6-phosphate. The reaction was initiated by adding 10 μL of glucose-6-phosphate dehydrogenase. NADPH was estimated spectrophotometrically (A_600nm_) as the oxidation of 2,6-dichlorophenolindophenol over 5–15 m.

Total glutathione was estimated spectrophotometrically using Ellman’s reagent, as previously described [36]. Glutathione was measured in total root tissue from 5 individual plants per treatment as well as from five separately pooled root tips (8–12 mm in length) from 8–10 plants.

### Protein electrophoresis

Immunoblotting was used to determine the effect of selenite treatment on various polypeptides in root tissue. Proteins were extracted in 50 mM Tris, pH 7.5, 100 mM NaCl, 0.5% triton-X, 2 mM dithiothreitol, 0.5 mM of the protease inhibitor PMSF, and heated for 10 min at 85°C. Unless otherwise stated, 20 μg of denatured protein were separated on reducing SDS-PAGE (10 or 12% acrylamide gels) and transferred to a PVDF membrane by electroblotting. Commercial antibodies for MnSOD, Csd1, Cox2, and AOX1 were obtained from Agrisera. Antibodies against APR2, SiR, Bip2 were used as described in previous studies [35]. Immunoreactive serum against UCP, protein bound lipoic acid, and MSRA4 were provided as generous gifts. Accumulation of ubiquitinated proteins in root tissue was tested in plants grown for 0, 1 and 3 days before being subsequently transferred to 20 mL of Hoagland’s media with or without 50 μM selenite containing 0.1% DMSO +/−100 μM MG132 for 8 hours. Ubiquitinated proteins were detected on 8% gels containing 50 μg of protein per lane, which reacted against ubiquitin antiserum (Santa Cruz Biotechnology) as previously described [19]. To detect polypeptides against the GGCT protein, a peptide sequence (GPEKEKLAMEYLERc) against the Arabidopsis homologue was synthesized (GeneScript, USA) and injected into rabbit. The antisera (1:1000) detected an immunoreactive protein that was about 25 kDA, which is in agreement with its predicted size in *B. rapa*. The immunoreactive protein was detected using alkaline phosphatase conjugated to the secondary antibody against rabbits.

### Visualization of ROS

The cell permeable fluorescent probe 2’ ,7’-dichlorodihydrofluorescein diacetate (H2DCFDA) was used to visualize ROS on day 3 or 7 to confirm that Se induced ROS accumulation in root tissue. Root tips (10–12 mm) were excised and placed in 1 mL of buffer containing 50 mM Tris, pH 7.5 and 10 μM H2DCFDA. Root tips were incubated on a rotating platform in the dark for 30 min, and subsequently washed 3 times in 50 mM Tris, pH 7.5, to remove residual H2DCFDA that would otherwise increase background fluorescence. H_2_DCFDA fluorescence optimally at Ex_492_/Em_525_, and was observed using a FITC filter.

Rapid accumulation of mitochondrial superoxide after 0, 1.5, 4, 16, 24, and 72 h of Se treatment was estimated using the probe MitoSox Red (Molecular Probes, Invitrogen) that selectively fluoresces in the presence of mitochondrial superoxide. Visualization of mitochondrial superoxide in 8–10 root tips was determined as stated above for H_2_DCFDA, except that root tips were incubated with 5 μM Mitosox Red for 15 min and the fluorescence (optimal Ex_510_/Em_580_) was visualized using a TRITC filter.

Cell viability and total glutathione were estimated nearly simultaneously in the same root tips from plants that were either untreated or treated with Se for 1 or 3 days. Root tips were incubated for 15 min in 1 mL of 50 mM Tris buffer, pH 7.5, containing 20 μM fluorescein diacetate and 25 μM monochlorobimane, which are indicative of cell viability and glutathione, respectively. Fluorescence of fluorescein diacetate (optimal Ex_495_/Em_515_) is dependent upon cell membrane integrity, and was estimated using a FITC filter set. Fluorescence of glutathione-conjugated monochlorobimane (optimal Ex_394_/Em_490_) was detected using a UV filter set.

Epifluorescence of all probes was detected on an Olympus BX51 microscrope. Images were captured using QCapture software, and are representative of 4–6 biological replicates.

### Enzymatic assays

Glucose-6-phosphate dehydrogenase activity in root tissue was determined as described [42], with some modifications. Root tissue (100 mg) from 6 different plants was ground in liquid nitrogen and extracted in protein extract buffer (50 mM Tris, pH 8, 100 mM NaCl, 0.5% triton-X, 5 mM beta-mercaptoethanol, 0.5 mM of the protease inhibitor PMSF). Supernatant was collected after centrifugation at 16,000 rpm. The enzyme assay was initiated by adding 10 μL of protein extract into 150 μL of reaction buffer (50 mM Tris, pH 8, 1 mM MgCl2, 100 μM of NADP, 0.4 mM of glucose-6-phosphate). Activity was determined spectrophotometrically based upon the reduction of NADP to NADPH at an absorbance of 340 nm.

Aconitase activity was measured in root tissue using an aconitase assay kit according to the manufacturer’s directions (Caymen Chemical, USA). Briefly, a non-denatured protein extract was collected as described above, and added to a reaction buffer containing 50 mM Tris, pH 7.4, sodium-citrate, NADP+, and isocitrate dehydrogenase. In this coupled-enzymatic reaction, citrate is isomerized via aconitase into isocitrate, which is then catalyzed by isocitrate dehydrogenase into ketoglutarate and NADPH. Aconitase activity was measured spectrophotometrically (A_340_) and was proportional to the reduction of NADP to NADPH.

The chymotrypsin activity of the proteasome was measured essentially as described [54]. Briefly, root tissue was ground in liquid nitrogen, and proteins were extracted in proteasome extraction buffer (50 mM potassium-phosphate buffer - pH 7.4, 5% (v/v) glycerol, 10 mM ATP, 5 mM beta-mercaptoethanol). Proteasome activity in root tissue was measured fluorometrically (Ex360/Em410) containing 3 μL of protein extract and 97 μL of reaction buffer (50 mM potassium-phosphate buffer, pH 7.4, 2 mM MgCl_2_, 1 mM ATP, 5 mM beta-mercaptoethanol) with 50 μM of the fluorogenic peptide Suc-LLVY-AMC in DMSO. The released fluorescence was recorded on a Glo-Max Multi spectrofluorometer (Promega, USA). Activity was determined after 30 min as the difference in increased fluorescence in reactions with or without MG132 (20 μM) to account for non-proteasomal release of AMC. Proteasome activity is expressed as the net changes of fluorescence (RFU min^−1^ μg protein^−1^) in the presence and absence of the proteasome inhibitor MG-132.

### Oxygen consumption and statistical analysis

Oxygen consumption in intact roots from 8 individual plants per treatment was measured using an Oxzilla Differential Oxygen Analyzer (Sable Systems International, USA). Excised root tissue (200–250 mg) was treated for 20 minutes in Hoagland’s solution with or without 1 mM potassium-cyanide to inhibit the cytochrome-c oxidase pathway. The root tissue was placed onto saturated filter paper with or without the inhibitor, and then incubated in a sealed 250 mL glass jar held at a constant temperature. Oxygen consumption was estimated as the difference in oxygen concentration in the reaction vessel after 15 min. Total respiration and the cynanide-resistant alternative respiration are expressed as the concentration of oxygen consumed-^1^ min^−1^ mg fresh weight of root tissue.

Statistical analyses were performed using the KaleidaGraph software program (Synergy Software), and included Student *t*-tests and analysis of variance (ANOVA).

### Availability of supporting data

All supporting data are included as additional files.

## Additional files

Additional file 1: Figure S1. The effects of selenite on cell viability, root length, and fresh weight to dry weight ratio in roots after 3 days of treatment with varied selenite concentrations. Additional file 2: Figure S2a-f. Selenite induces stress but does not alter photosynthetic parameters after 7 days of treatment. Additional file 3: Figure S3. Accumulation of total selenium and sulfur in plants treated with or without selenite for 7 days. Shown are the mean and SE from 5 different plants. Lowercase letters and uppercase letter represent a significant difference between treatments in root and shoot tissue, respectively (p < 0.05). Additional file 4: Figure S4. Amino acid sequence alignment of GGCT2; 1 and GGCT2; 2 in *Brassica rapa* and *Arabidopsis thaliana*.

## Abbreviations

ROS: Reactive oxygen species
Se: Selenium
TCA: Tricarboxylic acid
AOX: Alternative oxidase
COX: Cytochrome c oxidase
GSH: Glutathione
H_2_DCFDA: 2’,7’-dichlorodihydrofluorescein diacetate
