# Supplementary material for: Selenite activates the alternative oxidase pathway and alters primary metabolism in Brassica napus roots: evidence of a mitochondrial stress response
Source: BMC Plant Biol. 2014 Sep 30;14:259. doi: 10.1186/s12870-014-0259-6 (PMC4189625; doi:10.1186/s12870-014-0259-6)
Supplement: Additional file 1: Figure S1. — The effects of selenite on cell viability, root length, and fresh weight to dry weight ratio in roots after 3 days of treatment with varied selenite concentrations. [file 12870_2014_259_MOESM1_ESM.pdf]

Additional File 1: Figure S1

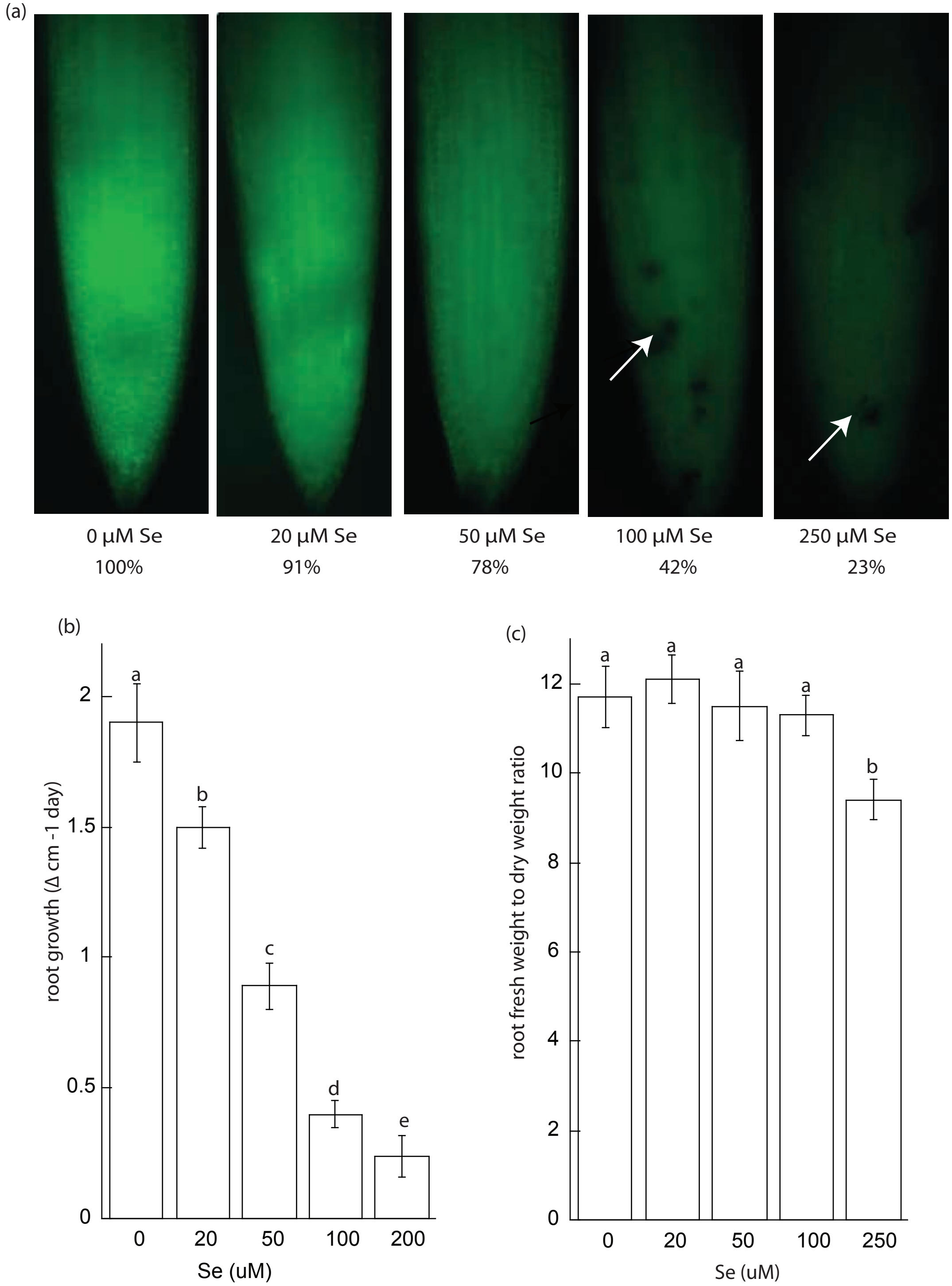

Additional file 1; Figure S1. The effects of selenite on cell viability (a), root length (b), and fresh weight to dry weight ratio in roots (c) after 3 days of treatment with varied selenite concentrations. Shown are the mean and SE from 10 different plants. Lowercase letters represent a significant difference between treatments ($p < 0.05$). Percentages correspond to percent viability relative to control. Arrows point to necrosis.
