# Supplementary figures and images for: Selenite activates the alternative oxidase pathway and alters primary metabolism in Brassica napus roots: evidence of a mitochondrial stress response
Source: BMC Plant Biol. 2014 Sep 30;14:259. doi: 10.1186/s12870-014-0259-6 (PMC4189625; doi:10.1186/s12870-014-0259-6)

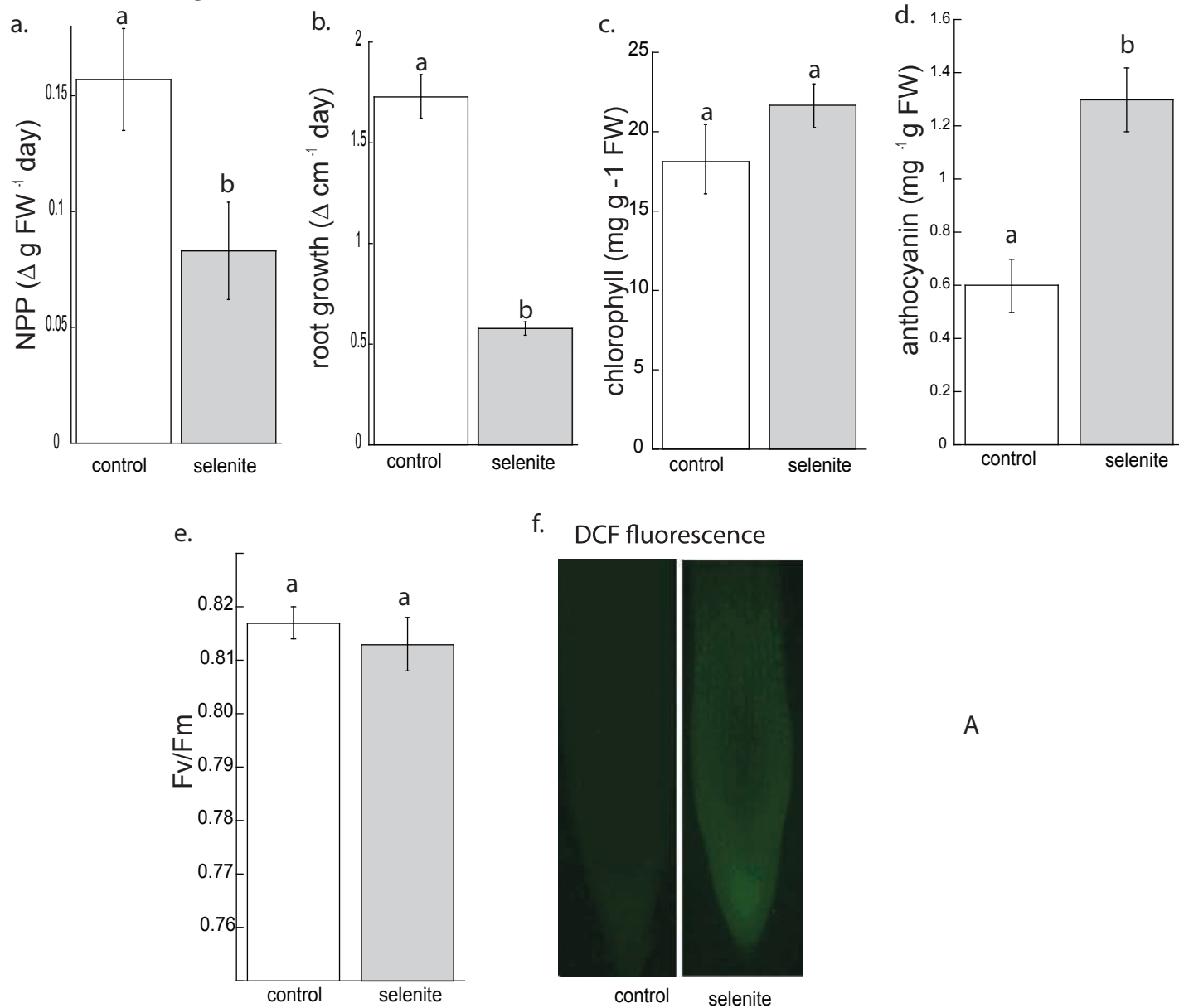

Supplement: Additional file 2: Figure S2a-f. — Selenite induces stress but does not alter photosynthetic parameters after 7 days of treatment. [file 12870_2014_259_MOESM2_ESM.pdf]

Additional File 3: Figure S3

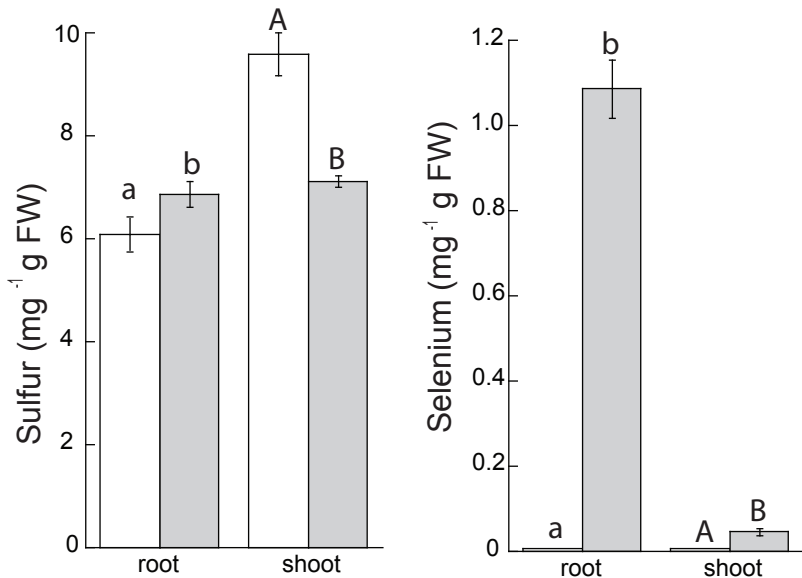

Supplement: Additional file 3: Figure S3. — Accumulation of total selenium and sulfur in plants treated with or without selenite for 7 days. Shown are the mean and SE from 5 different plants. Lowercase letters and uppercase letter represent a significant difference between treatments in root and shoot tissue, respectively (p < 0.05). [file 12870_2014_259_MOESM3_ESM.pdf]
