# Supplementary material for: Selenite activates the alternative oxidase pathway and alters primary metabolism in Brassica napus roots: evidence of a mitochondrial stress response
Source: BMC Plant Biol. 2014 Sep 30;14:259. doi: 10.1186/s12870-014-0259-6 (PMC4189625; doi:10.1186/s12870-014-0259-6)
Supplement: Additional file 4: Figure S4. — Amino acid sequence alignment of GGCT2; 1 and GGCT2; 2 in Brassica rapa and Arabidopsis thaliana. [file 12870_2014_259_MOESM4_ESM.pdf]

```
At_GGCT2;2      MVMWVFGYGSLVWNPGFHYDEKVLGFIKGYKRVFDLACIDHRGTPEHPARTCTLEKAEEA 60
Br_GGCT2;2      MVMWVFGYGSLVWNPGFHYDEKVLGFIKGYKRVFDLACIDHRGTPEHPARTCTLEIDEEA 60
At_GGCT2;1      MVLWVFGYGSLIWNPGFDFDEKLIGYIKDYKRVFDLACIDHRGTPEHPARTCTLEQSTGA 60
Br_GGCT2;1      MVLWVFGYGSLIWNPGFDFDEKLIGYIKDYKRVFDLGCIDHRGTPEHPARTCTLEESTGA 60
                **:********:*****.:***::*:**.*******.******************    *

At_GGCT2;2      ICWGTAFCVRGGPEKERLAMEYLERRECEYDLKTSVDFYKEDDP---LKPAVTGVIVFTS 117
Br_GGCT2;2      ICWGTAFCVRGGPEEERLAMEYLERRECEYDLKTCVDFYKEDDP---LNPAVTGVMVFTS 117
At_GGCT2;1      ICWGAAYCVRGGPEKEKLAMEYLERRECEYDSKTLVEFYTENDT---STPIVTGVIVFTS 117
Br_GGCT2;1      ICWGAAYCVRGGPEKEKLAMEYLEGRECEYDSKTLVEFYTETDTTKQSKPILTGVIVFTS 120
                ****:*:*******:*:******* ****** ** *:**.* *.    .* :***:****

At_GGCT2;2      TPDKVSNKYYLGPAPLEDMARQIATANGPCGNNRDYLFLLEKAMHDIGHEEDYVIELANE 177
Br_GGCT2;2      TPDKVSNKYYLGPAPLEDMARQIATANGPCGNNRDYLFLLEKAMHDIGHEEEYVIELANE 177
At_GGCT2;1      TPDKVSNKYYLGPAPLEEMARQIATASGPCGNNREYLFKLEKAMFDIEHEEEYVIELANE 177
Br_GGCT2;1      TPDKLSNKYYLGPAPLEEMAMQIATASGPCGNNREYLFKLEKAMHDIEHEDEYVIELANE 180
                ****:************:** *****.*******:*** *****.** **::********

At_GGCT2;2      VRKVLAESSTKKVTPVKESRASRVANK--SKNNVPTAHQILPHHPEAVATTI 227
Br_GGCT2;2      VRKVLAE----KVSPVKESRASPVAKK--SKSNVPTAHQILPHQPEAVATTL 223
At_GGCT2;1      VRKQLDLP--EEVKALLKPIVSHVSVK--SQAHVSTRQRVFAS--------- 216
Br_GGCT2;1      VRKHLDLP--KEVKALLKPVVSRVSIKSHSQAHASTLQRVFA---------- 220
                *** *      :*..: :. .* *: *  *: :..* ::::.
```

Additional file 4: Figure S4 Amino acid sequence alignment of GGCT2;1 and GGCT2;2 in *Brassica rapa* (Br) and *Arabidopsis thaliana* (At). The multiple sequence alignmnet was made using CLUSTAL 2.1. Sequence in red represents the peptide sequence used to make an antiserum reactive against the GGCT protein.
